# Reaction Pathways in Catechol/Primary Amine Mixtures: A Window on Crosslinking Chemistry

**DOI:** 10.1371/journal.pone.0166490

**Published:** 2016-12-08

**Authors:** Juan Yang, Vittorio Saggiomo, Aldrik H. Velders, Martien A. Cohen Stuart, Marleen Kamperman

**Affiliations:** 1 Laboratory of Physical Chemistry and Soft Matter, Wageningen University, Wageningen, the Netherlands; 2 Laboratory of BioNanoTechnology, Wageningen University, Wageningen, The Netherlands; Brandeis University, UNITED STATES

## Abstract

Catechol chemistry is used as a crosslinking tool abundantly in both natural organisms (e.g. mussels, sandcastle worms) and synthetic systems to achieve the desired mechanical properties. Despite this abundance and success, the crosslinking chemistry is still poorly understood. In this study, to simplify the system, yet to capture the essential chemistry, model compounds 4-methyl catechol and propylamine are used. The reaction of 4-methyl catechol (2 mM) with propylamine (6 mM) is carried out in the presence of NaIO_4_ (2 mM) in 10 mM Na_2_CO_3_ aqueous solution. A variety of spectroscopic/spectrometric and chromatographic methods such as ^1^H NMR, LC-MS, and UV-VIS are used to track the reaction and identify the products/intermediates. It is found that the crosslinking chemistry of a catechol and an amine is both fast and complicated. Within five minutes, more than 60 products are formed. These products encompass 19 different masses ranging from molecular weight of 179 to 704. By combining time-dependent data, it is inferred that the dominant reaction pathways: the majority is formed via aryloxyl-phenol coupling and Michael-type addition, whereas a small fraction of products is formed via Schiff base reactions.

## Introduction

**Fig 1:**
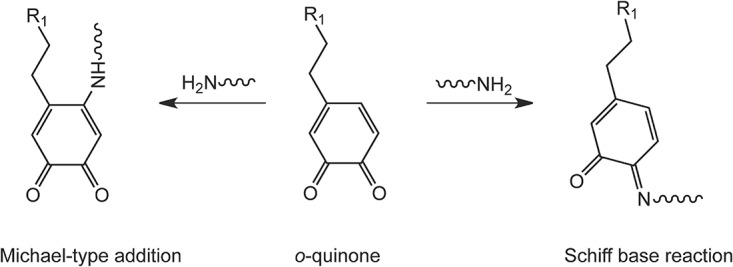
Reaction of amines with *o*-quinones via Michael-type addition or Schiff base reaction

Catecholic compounds are widely distributed among natural animal and plant systems[1–3]. One famous example of a catecholic compound is 3,4-dihydroxyphenylalanine (DOPA). When exposed to air, DOPA is prone to oxidation. The formed *o*-quinones may further react with a variety of nucleophiles in various pathways to form crosslinks[3,4]. A well-known nucleophile is the amine that may react with *o*-quinones to form adducts either by Michael addition or Schiff base reaction (Fig 1)[4].

The reaction between catechols and amines is of vital importance in natural biological processes, such as the crosslinking of adhesive proteins by marine organisms[5], the formation of cytoskeleton by insects[6] and the biosynthesis of melanin[7]. For instance, in blue mussels, both DOPA and *L*-lysine are present in large quantities in mussel adhesive foot proteins (mfps)[8]. It has been proposed that the -NH_2_ group in lysine side chains in mfps may react with the carbonyl groups of *o*-quinones to form Schiff-base adducts[9]. The reaction between catechols and amines, together with all other possible reactions involving catechols, contribute to the fast solidification of freshly secreted mfps to form a tough and robust cuticle[9]. Similar *o*-quinone-amine adducts have also been identified in insect sclerotization. By isolation and proteolysis of the natural proteins from beak cutouts of squid Dosidicus gigas, Waite et al. has identified the presence of multimers (dimers, trimers and tetramers) of catechol–histidine adducts[11,10].

The catechol-amine chemistry occurring in natural organisms has attracted much attention in material science[11–13]. Several research groups have developed water-soluble polymers that form a gel or water-resistant film upon reaction between a catechol and an amine. For instance, Lee et al. synthesized a poly(ethylene glycol) polymer containing both catechol and amine functional groups[11]. Upon the reaction between the catechol and amine, the water-soluble polymer formed rigid hydrogels in one minute. Similarly, Xu et al. prepared a multilayered film by alternatively immersing a substrate in aqueous solutions of poly(acrylic acid-dopamine) (PAA-dopamine) and poly(allyamine hydrochloride) (PAH), respectively. After triggering the reaction of catechol and amine, a stable and robust film was obtained[13].

Despite the extensive literature on material development using catechol-amine reactions, the possible mechanisms remain unclear[14]. Several mechanistic studies have been performed to gain more fundamental understanding. For instance, Kodadek et al. studied the reaction between catechol and amines using two peptide nucleic acids[15]. They found that crosslinks were formed through Michael addition between amines with *o*-quinones. In addition, they formulated two premises that should be fulfilled for the reaction to proceed: i) close proximity of *o*-quinone intermediate and amine nucleophiles, and ii) the presence of sodium periodate. Waite et al. also studied the reaction mechanism by using natural decapeptides derived from mfp1, which contains both DOPA and lysine. They started the reaction by adding oxidants such as sodium periodate, or tyrosine. By using HPLC, MALDI-TOF, and amino acid analysis, they observed that lysine is likely to contribute to intramolecular crosslinking with DOPA, rather than to intermolecular crosslink formation[9]. These studies showed that the reaction mechanism differs strongly depending on the specific reaction conditions.

The mechanistic studies mentioned above have given valuable insights in the crosslinking chemistry of catechols and amines. However, a thorough understanding of the mechanisms based on detailed product identification is still missing, resulting in uncontrollable crosslinking in many bio-mimetic materials. The difficulty in exploring the crosslinking chemistry in natural systems is related to the complexity of full-length proteins, and to ambiguities in tyrosine oxidation to DOPA or *o*-quinones. Therefore, in this study, instead of using proteins, we used small model compounds to study the crosslinking chemistry. The model compounds are 4-methyl catechol (4MC) and propylamine (PA). Sodium periodate (1 mM) and sodium carbonate aqueous solution (10 mM) were used as oxidant and solvent, respectively. By using HPLC and LC-MS, we found that the reaction of 4MC and PA is very fast, and more than 60 products are formed in less than five minutes. Among these products, the majority is mainly from Michael-type addition and phenol coupling. Additional products are formed by Schiff-base reaction.

## Experimental Section

### Synthesis of model compound

**Fig 2:**
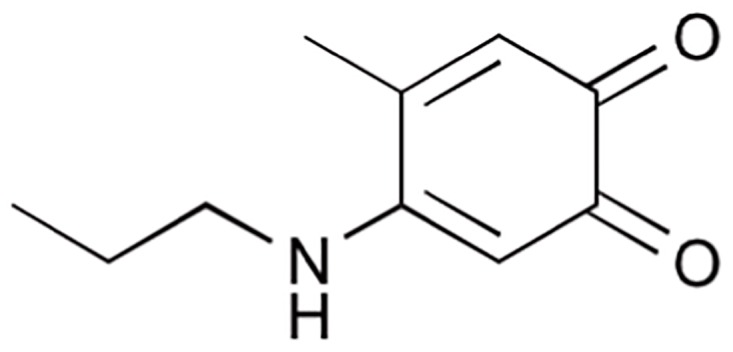
Chemical structure of 4-propylamino-5-methyl-*o*-quinone

The synthesis of the model compound 4-propylamino-5-methyl-*o*-quinone (Fig 2) was adapted from the procedure reported by Suyama, et al. Sodium iodate (6 g, 30 mmol) in 60 ml of milli-Q water was added slowly to a mixture of 4MC (4.97 g, 40 mmol) and PA (2.42 g, 41 mmol) in 80 ml of acetic acid. After one hour of reaction in an ice-bath, the reaction mixture was slowly added to 500 ml cold milli-Q water, and followed by filtration. The solid material was dissolved in 200 ml acetonitrile, and extracted three times by 300 ml *n*-hexane. The fitrate was washed three times with 500 ml *n*-hexane. All hexane layers were collected, and concentrated using rotary evaporation. Afterwards, the red solution was collected and separated by silica gel column chromatography with a *n*-hexane/diethyl ether (8:2, v/v) mixture as eluent. Five fractions were collected and further subjected to HPLC analysis.

### Sample preparation for HPLC and LC-MS

Reactions mixtures for HPLC and LC-MS measurements were prepared by adding 4MC (1.24 mg, 10 μmol), PA (2.46 μl, 30 μmol), and sodium periodate (2.14 mg, 10 μmol) to 5 ml of sodium carbonate (10 mM) aqueous solution.

### Analytical HPLC analysis

Analytic HPLC was carried out on Hewlett Packard 1100 systems coupled to UV detector and the data were processed using HP Chemstation software. Separations were performed using a reverse-phase HPLC column (Alltima C18-5u, 4.6 mm, 250 mm) after injecting 10 μl of the sample. Except where otherwise stated, the chromatography was carried out using UV detection at 254, 260 and 280 nm. A flow rate of 1 ml min^−1^ was used. The mobile phase consisted of a mixture of acetonitrile and milli-Q water (both containing 0.1% formic acid), using the following gradient: (0 min: 5% CH3CN; 13 min: 45% CH_3_CN; 23 min: 95% CH_3_CN; 32 min: 95% CH_3_CN; 35 min: 5% CH_3_CN; 43 min: 5% CH_3_CN).

### LC-MS analysis

LC-MS was performed using a HPLC-MS from Thermo Scientific, Finnigan LXQ series. Analyses were performed using a reversed-phase HPLC column (Alltima C18-5u, 4.6 mm, 250 mm) injecting 10 μL of the sample. A flow rate of 1 ml min^−1^ was used. A splitter was used, 0.2 ml min^−1^ entered the MS detector and 0.8 ml min^−1^ was disposed off. The mobile phase consisted of a mixture of acetonitrile and milli-Q water (both containing 0.1% formic acid), using the following gradient: (0 min: 0% CH_3_CN; 13 min: 55% CH_3_CN; 23 min: 5% CH_3_CN; 32 min: 5% CH_3_CN; 35 min: 95% CH_3_CN; 43 min: 95% CH_3_CN). Positive ion mass spectra were acquired in ultrascan mode using electrospray ionization. MS analysis was performed using electrospray ionization (ESI) and detection in the positive mode, with a source voltage of 5.0 kV and an ion transfer tube temperature of 275°C.

### Dynamic light scattering (DLS)

Dynamic light scattering was used to monitor the formation of high molecular weight products between 4MC and PA. Briefly, a reaction mixture containing 2mM 4MC, 3 mM PA, and 2 mM sodium periodate in 5ml of 10 mM sodium carbonate aqueous solution was filtered through a 0.45 μm polyethersulfone filter. Light scattering was performed using an ALV dynamic light scattering instrument with a Cobolt Samba- 300 DPSS laser (300 mW) operating at a wavelength of 532 nm and an ALF-5000/60X0 multiple *τ* digital correlator. A refractive index matching bath of filtered cis-decalin surrounded the cylindrical scattering cell. All measurements were performed at a fixed angle *θ* of 90°, corresponding to a scattering vector $\text{q}=\frac{4\pi n}{\lambda }\text{sin}\frac{\theta }{2}$ ~ 0.02 nm^-1^, where *n* is the refractive index of the solvent (water). The temperature was kept constant at 20°C using a Haake F3-K thermostat.

### ^1^H NMR spectroscopy

^1^H NMR of the sample was performed in D_2_O on a Bruker AMX-400 spectrometer (400 MHz) at 25°C.

### UV-VIS spectroscopy

UV-visible characterization was performed on a Shimadzu UV-2600 spectrophotometer. The samples were measured using a wavelength scan range of 200–600 nm in a quartz cuvette.

## Results and Discussion

### Reaction system design

The crosslinking chemistry of catechols and amines is complicated and the mechanisms are still under dispute. Generally, it is accepted that the nucleophilic amines attack oxidized catechols by either Michael-type addition or forming a Schiff base. However, a detailed understanding of the identities of the products is still missing. In this study, we used several techniques to identify the products of catechol-amine crosslinking reactions. The proper choice of reaction system and reaction conditions is important. In material science, most often high molecular weight molecules are linked together with catechol and amine reactions. In this mechanistic study, ^1^H NMR is one of the techniques we used. It is thus desirable to keep the system as simple as possible to avoid possible peak broadening and overlapping. For this reason, we chose the simplest form of catechol and α-amino derivatives, i.e. 4MC and PA, respectively. By using these two model compounds, we expect to capture the essential chemistry between catechol and amine. This essential chemistry would facilitate a better understanding of the catechol-amine crosslinking mechanism in catechol-containing polymer systems. To precisely identify the reaction products, the reaction conditions should fulfill the following requirements. Firstly, it is generally accepted that, for primary amines to work effectively as nucleophiles, they should not be charged[16]. Therefore, pH is an essential parameter. In our case, since the pKa of PA is 10.71, we maintained the pH of the reaction medium around 11 using 10 mM aqueous sodium carbonate solution as the solvent. Secondly, at high pH, as reported, the reaction rate of catechol oxidation is the rate-determining step that affects further reactions. Therefore, we add an oxidant NaIO_4_ (0.5 equivalent to catechol) to form enough *o*-quinones[17]. Thirdly, generally, the reaction between amines and *o*-quinones is slightly slower than that of aryloxyl coupling[18]. To increase the likelihood of reacting *o*-quinones with amines, we set the PA/4MC molar ratio to 3. This excess of amines with respect to catechols may well represent the scenario in the common synthetic polymer systems, in which the polymer usually contains more amines than catechols. Finally, at high temperatures *o*-quinones may react with amines via Strecker degradation to produce aldehydes and 2-aminocarbonyl compounds[19]. However, the crosslinking of catechols and amines for polymer systems usually proceeds at ambient conditions[16]. Therefore, we set the reaction temperature at 20°C.

### ^1^H NMR analysis

**Fig 3:**
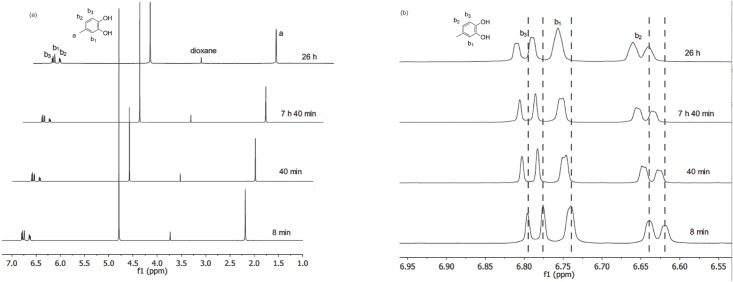
^1^H NMR spectra monitoring the reaction of 4MC in 10mM Na_2_CO_3_/D_2_O with time. Dioxane was used as a reference. (a) Full overview of the ^1^H NMR spectra (b) ^1^H NMR spectra in the aromatic region.

**Fig 4:**
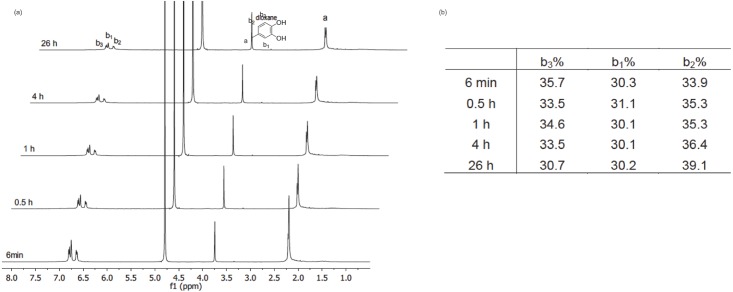
a) ^1^H NMR spectra monitoring the reaction of 4MC and NaIO_4_ in 10 mM Na_2_CO_3_/D_2_O with time. The molar ratio between 4MC and NaIO_4_ is 0.5. Dioxane was used as a reference. b) Relative integrated peak area of b_1_, b_2_, and b_3_ with respect to the total aromatic protons.

The reaction of 4MC, PA and NaIO_4_ in 10 mM Na_2_CO_3_ aqueous solution was monitored by ^1^H NMR measurements with the concentration of 4MC as 213 mM, and the molar ratio of 4MC/PA/ NaIO_4_ = 1/3/0.5. To have a clear understanding of the role of PA, we also studied two reference reactions: 4MC in 10 mM Na_2_CO_3_ (aq) and 4MC in the presence of NaIO_4_ in 10 mM Na_2_CO_3_ (aq). For 4MC in 10 mM Na_2_CO_3_ (aq), the solution turned pink immediately, and after 26 h, no precipitates were observed. The pinkish color indicates that some reactions might have been taking place. This indication is supported by the observation in Fig 3(b), that the aromatic proton peaks are gradually shifted downfield. The shift might be due to the formation of *o*-quinones, in which the electron-withdrawing property of the carbonyl groups has a “deshielding” effect on the protons. Moreover, as shown in Fig 3(a), little change is observed in the intensity of the methyl peaks (δ = 2.2 ppm) and the aromatic peaks (δ = 6.5–6.8 ppm) over time (Fig 3(a)). The little change indicated that the reaction proceeded at a very slow rate. The slow rate might be due to relatively slow catechol oxidation, which has been identified to be the rate-determining step under these conditions[17]. To speed up the reaction, NaIO_4_ was added, and the reaction solution immediately turned dark red. In less than one hour, dark red precipitates were observed, which could not be detected by liquid ^1^H NMR. However, we may rule out the possibility that these precipitates being the reduction intermediates/products of NaIO_4_, which has been proposed to bear a negative charge and should be water-soluble[4]. As shown in Fig 4, the aromatic peaks (δ = 6.5–6.8 ppm) and methyl peaks (δ = 2.2 ppm) decreased significantly over time, together with the appearance of red precipitates, indicating the formation of aggregates. Meanwhile, a very broad peak at around δ = 1.5 ppm emerged after 6 min of reaction (See ESI, S1 Fig)), and the integration of the peak increased over time. This intensified broad peak might be due to the formation of water molecules released from the coupling reaction of (oxidized) 4MC, which subsequently would form catechol-catechol oligomer-like structures. In addition, the ratio between the integrated area of aromatic protons (b_1_+b_2_+b_3_) and the methyl group (a) decreased overtime, indicating that the protons on the aromatic ring reacted overtime. If we take a closer look at the integrated area of the aromatic protons, b_1_ does not change overtime; while b_3_ showed a decreasing trend and b_2_ increased overtime. This trend is attributed to the reactivity of position b_3_ by phenol coupling. All things considered, addition of NaIO_4_ has significantly increased the reaction rate of catechol oxidation/crosslinking.

**Fig 5:**
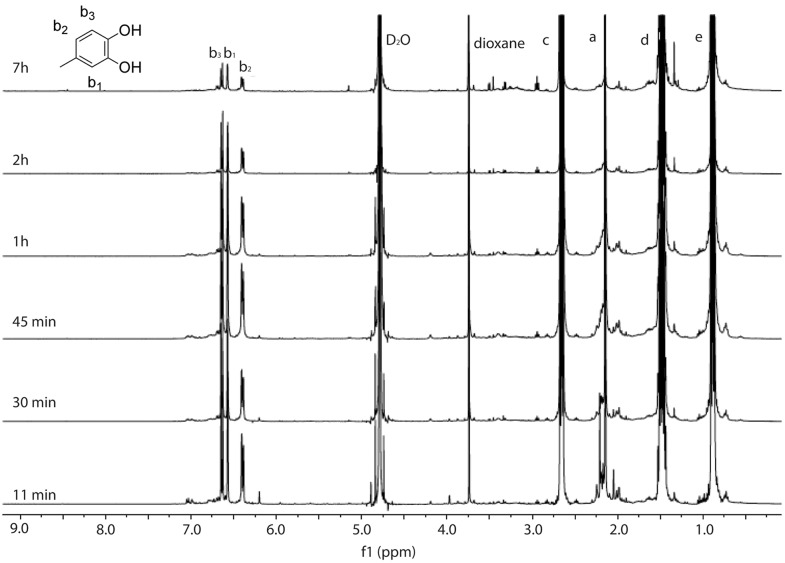
^1^H NMR spectra monitoring the reaction of 4MC, PA and NaIO_4_ in 10 mM Na_2_CO_3_/D_2_O with time. The molar ratio between 4MC, PA and NaIO4 is 1:3:0.5. Dioxane was used as a reference.

Fig 5 shows representative ^1^H NMR spectra of the reaction of 4MC, PA and NaIO_4_ in 10 mM Na_2_CO_3_ aqueous solution. As soon as the reagents were mixed, the solution turned dark red. After one hour of reaction, dark red precipitates appeared. As shown in Fig 5, the peaks related to 4MC, namely, the aromatic peaks at *δ* of 6.3–6.75 ppm, and methyl peaks at *δ* = 2.1 ppm both decreased significantly in two hours. Moreover, broad peaks close to *δ* = 2.1 ppm and *δ* = 7.0 ppm appeared within 11 min, and decreased again over time. The peaks related with PA, namely, *δ* of 0.9, 1.48, and 2.65 ppm also decreased with time. Meanwhile, three small peaks (marked in red) appeared. These three peaks were shifted downfield and showed similar shapes as the peaks related with the alkyl groups of PA (positions e, d, and c). The integrated area ratio of these peaks is 3:2:2, which is the same ratio as the PA protons. This observation might indicate that more pronounced reactions taking place under current reaction conditions. However, a detailed understanding on the reaction is still missing.

Collectively, the ^1^H NMR measurements suggest that oxidation of 4MC at high pH is slow and sodium periodate is needed to speed up the catechol oxidation. The oxidized 4MC may react with PA to form catechol-amine adducts. However, ^1^H NMR could not detect the precipitates observed in the reaction mixture, and additional techniques are needed to identify the reaction products.

### UV-vis measurements

UV-vis spectroscopy is a technique often used in catechol-related literature to follow catechol-involving reactions. Therefore, in this study, to gain additional insights into the reaction of 4MC with PA, we monitored the reaction mixture over time using UV-vis spectroscopy. Two reference reactions were also studied. The concentration of 4MC is 1 μM, and the ratio of 4MC/PA/ NaIO_4_ = 1/3/0.5.

**Fig 6:**
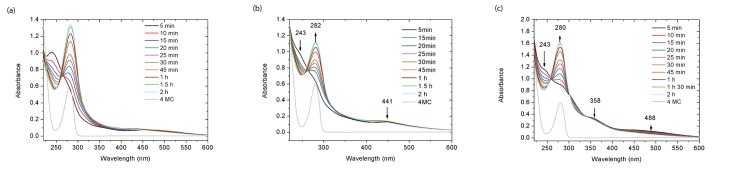
UV-vis absorbance monitoring the reaction under different conditions: a) 4MC in 10 mM Na_2_CO_3_ aqueous solution; (b) 4MC and NaIO_4_ in 10 mM Na_2_CO_3_. The molar ratio between 4MC and NaIO_4_ is 0.5; (c) 4MC, PA and NaIO_4_ in 10 mM Na_2_CO_3_. The molar ratio between 4MC, PA and NaIO_4_ is 1:3:0.5.

As shown in Fig 6(a), 4MC in Milli-Q water showed a single characteristic absorbance peak at *λ*_*max*_ = 280 nm, which is attributed to unreacted catechols. Upon adding 4MC to 10 mM Na_2_CO_3_, the transparent aqueous solution turned pink immediately. The characteristic peak of catechol at *λ*_*max*_ = 280 nm disappeared, along with the appearance of two new peaks at *λ*_*max*_ = 240 and 448 nm. These two peaks might reveal the formation of *o*-quinones by catechol oxidation[9]. As the reaction proceeded, the peaks at *λ*_*max*_ = 240 nm and *λ*_*max*_ = 448 nm decreased, with the simultaneous appearance of peaks at 277 nm (after 15 min). These changes suggest that the *o*-quinones react further to form other intermediate products (see S6 Fig). The new peaks at 277 nm gradually shifted to 283 nm within 50 min, along with a gradual intensity increase, which might be an indication of the transformation from intermediate products to di-DOPA crosslinks[9].

Similar UV-vis spectra were also obtained for the reaction of 4MC in 10 mM Na_2_CO_3_ aqueous solution in the presence of NaIO_4_, as shown in Fig 6(b). The difference with the reaction without NaIO_4_ is that the peak at around *λ*_*max*_ = 240 nm was hardly visible at 5 min, which means that *o*-quinones had already reacted further on that time scale. Apart from that, the spectra showed similar changes as those in Fig 6(a). More specifically, the peaks at *λ*_*max*_ = 441 nm decreased with time; and a new peak at *λ*_*max*_ = 277 nm appeared (after 20 min), which gradually shifted back to *λ*_*max*_ = 282 nm (after 1.5 h) and intensified over time. These changes also indicate that the *o*-quinones reacted further to form other products. It is unclear, however, what the reason is of the longer time taken for the appearance and shift of the peak at *λ*_*max*_ = 277 nm. Perhaps, it is related to the increased *o*-quinone concentration on the kinetics of further reactions. Nevertheless, the presence of NaIO_4_ did not alter the essential chemistry of the 4MC reaction.

The UV-VIS spectrum of 4MC and PA together in the presence of NaIO_4_ in 10 mM Na_2_CO_3_ aqueous solution is shown in Fig 6(c). By comparing to the spectra for 4MC alone, or 4MC with NaIO_4_, some similarities can be seen, i.e., the initial disappearance of the characteristic catechol peak at *λ*_*max*_ = 280 nm; and the gradual peak shift from 277 nm (observed at 15 min) to 280 nm (observed after 35 min). These similarities suggest that in all cases oxidation of catechol to *o*-quinones proceeds immediately. In contrast, new peaks appeared at *λ*_*max*_ = 358 and 488 nm, which gradually decreased somewhat over time, and concurrently, the intensity of the peak at 280 nm increased significantly. These changes indicate the occurrence of subsequent reaction of *o*-quinones. The increased absorbance at wavelengths higher than 500 nm has been associated in the literature with the formation of products from Michael type addition of catechol and amines[20].

### HPLC

**Fig 7:**
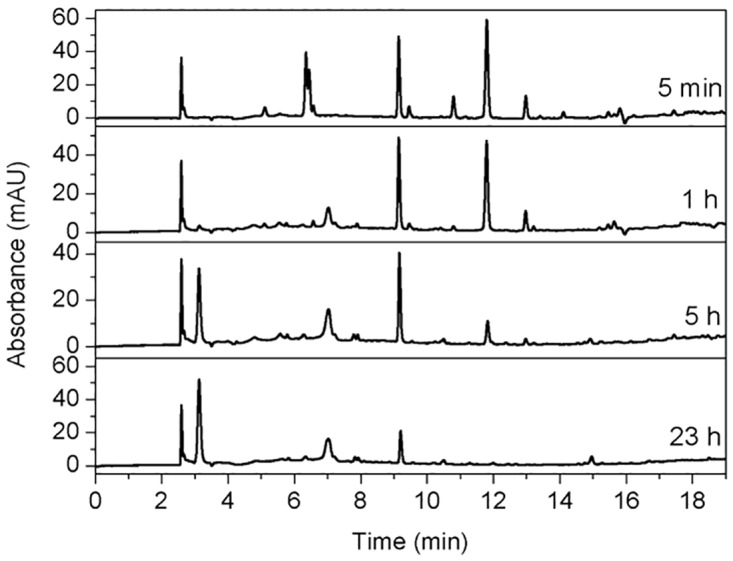
HPLC-UV chromatograms (monitored at 254 nm) of the reaction mixture of 4MC, PA, and NaIO_4_ with time. The ratio of 4MC, PA and NaIO_4_ is 1:3:0.5.

Collectively, the ^1^H NMR and UV-VIS data suggest that 4MC reacts differently in the presence of PA than without. It is generally accepted that multiple reactions of 4MC and PA may take place. The presence of multiple reactants/products brings difficulty in interpreting the UV-vis data. Therefore, in the following, we use HPLC to separate the reactants/products from the reaction mixture, and to study the time dependence of the reaction (Fig 7).

As shown in Fig 7, the reaction proceeds very fast, as evidenced by multiple peaks already emerging in 5 min after mixing the reagents. Under the chromatographic conditions employed, NaIO_4_ eluted from the column almost immediately, which is assumed to quench the reaction effectively. There are no peaks related to unreacted 4MC, which should, as we checked, elute at a retention time of 12.2 min, in Fig 7, suggesting that all 4MC has undergone oxidation. Since PA is not detected by UV, and 4MC has already reacted to *o*-quinones, the peaks shown in Fig 7 must be considered as evidence of products/intermediates. The peaks at retention times around 6.34 and 6.5 min, as shown in the profile after 5 min of reaction, disappeared after one hour of reaction, indicating that the corresponding compounds must be highly reactive intermediates. After one hour of reaction, two peaks at retention times of 3.1 and 7.0 min started to emerge, and they increased until the longest recorded reaction time of 24 h. Aside from these two peaks, all the other peaks decreased with time. Therefore, the elution patterns of 5 min and one hour already represent the majority of the products/intermediates. Therefore, these two chromatographic profiles were further characterized by mass spectrometry.

### LC-MS analysis for peak identification

**Fig 8:**
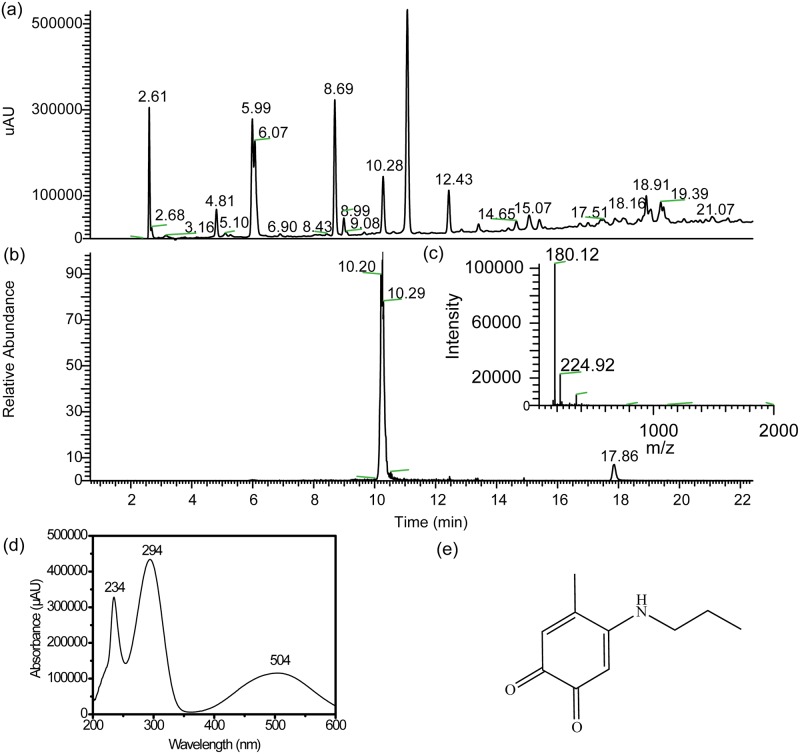
a) LC-MS chromatograms (monitored at 254 nm) of the reaction mixture of 4MC, PA, and NaIO4 over time. The ratio of 4MC, PA and NaIO4 is 1:3:0.5. b) Extracted ion chromatogram (positive ion mode) corresponding to PMB (m/z = 179.5–180.5) c) ESI-MS spectra [m/z 50–2000] summed over the 10.00–10.53 min retention time window; d) Extracted UV spectra corresponding to the 10.00–10.53 min retention time e) Proposed representative chemical structure of product.

To identify the possible intermediates/products in HPLC, we obtained mass spectra for the observed peaks in HPLC using mass spectrometry. Here one should note that all the peaks in the elution profile of Fig 7 are slightly shifted due to equipment differences as shown in Fig 8. However, this shift does not affect the chemistry of the reaction.

**Fig 9:**
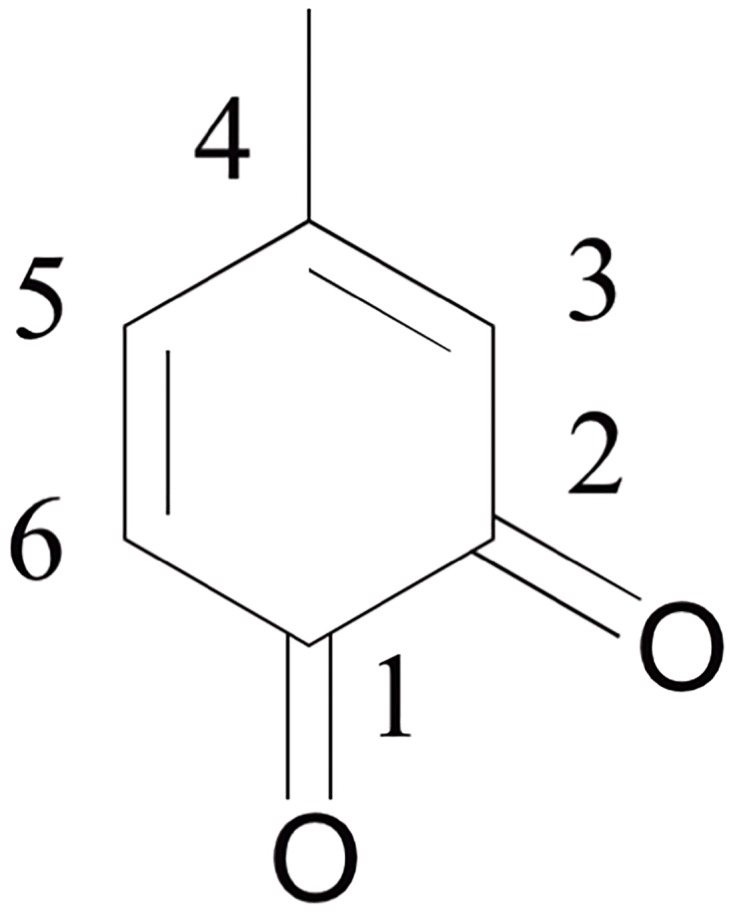
Chemical structure of 4-methyl-*o*-benzoquinone with numbers indicating the position of C atoms on the aromatic ring.

In general, as discussed previously in Fig 1, the reaction between oxidized catechol and amine in basic aqueous conditions can proceed via two pathways: 1) Michael-type addition of amine to *o*-quinone; 2) Schiff base reaction of amine to *o*-quinone. Moreover, *o*-quinones may also react with catechols via dismutation to form catechol-catechol crosslinks. As seen in Fig 9, the phenol radical coupling generally takes place at positions 3, 5, 6, and possibly at positions 1 and 2. Coupling of two catechols would lead to a mass loss of 2 Da [9] at position 3 5 and 6 and a mass loss of 16 Da at position 1 or 2. For Michael-type addition, the nucleophilic amine, generally attacks position 3 or 5 on the *o*-quinone by 1,6-addition, and position 6 by 1,4- addition. Therefore, based on the total mass of *o*-quinone and PA, the mass of the final product would decrease by 2 Da[9]. For Schiff-base reactions, the substitution usually takes places at positions 1 or 2, resulting in a mass loss of 18 Da based on the total mass of *o*-quinone and PA. Therefore, the mass of the prominent peaks *m* in the chromatogram should be related to the mass of 4MC (*m*_*4MC*_), 4-methyl-o-benzoquinone (*m*_*4MQ*_) and PA (*m*_*PA*_) by the Eq 1:

$$m=n_{1}m_{4MC}+n_{2}m_{PA}+n_{3}m_{4-MQ}-2n_{4}-18n_{5}$$ (1)

in which *n*_*1*_, *n*_*2*_, *n*_*3*_, *n*_*4*_ and *n*_*5*_ are integers. *n*_*4*_ is related to the coupling in Michael-type addition or phenol-phenol coupling, *n*_*5*_ is related to the Schiff base formation. Based on this equation and the mass we obtained from mass spectrometry, we can postulate the structures corresponding to the peaks.

In total, we have identified 19 m/z values for all these peaks. Here we will elaborate on a few examples for the major peaks at different retention times. As shown in Fig 8, the peak at retention time of 10.28 min showed a strong signal in the mass spectrum (Fig 8(c)) at m/z = 180.10. This value matches the structure of 4-*n*-propylamino-5-methyl-1,2-benzoquinone (PMB) (Fig 8(e)). To further verify the structure of the compound, we attempted to isolate PMB by collecting the fraction with retention time 10.28 min from HPLC elution. However, after lyophilization of the collected fractions, the HPLC chromatogram of the sample showed a different retention time. Moreover, the ^1^H NMR spectrum of the fraction did not match the structure, indicating that the compound had degraded. The degradation might be due to two possible reasons. Firstly, during lyophilization, formic acid from the HPLC elution became more concentrated over time, leading to higher acidity, which may have hydrolyzed PMB. Secondly, PMB may have further reacted to form higher molecular weight products due to its high reactivity. If the first reason is the most important, it might be possible to obtain pure PMB via a different route for NMR structural analysis. For this reason, we tried to synthesize PMB directly from the reaction of 5-methyl-1, 2-benzoquinone and PA in acetic acid, which was modified from a reported protocol[21]. Although we kept the pH of the reaction medium slightly acidic to prevent PMB hydrolysis, this strategy proved to be unsuccessful. The product is unstable and dark brown precipitates were observed when the product was concentrated in organic solvent (e.g. hexane, diethyl ether) during purification. The instability has been reported for a similar compound, 4-*n*-butylamino-5-methyl-1,2-benzoquinone[22]. Additionally, the UV-vis spectra of eluted PMB showed distinct peaks at 294, and 504 nm, which matched the UV-vis spectra of the amine-catechol adduct reported in literature [21,22]. Nevertheless, one might argue that we did not observe these two distinct peaks in Fig 6(c). We ascribed this to the fact that Fig 6(c) presents collective spectra of so many products/intermediates in the reaction mixture, that peaks of individual species are obscured.

S2 Fig shows the complete structural details of the possible product at retention time 11.07 min. As shown in S2(b) Fig, the mass m/z = 440.31 was detected at more than one retention time, e.g., 10.00, 11.00, 19.33 min, indicating that multiple products/isomers with m/z of 440.31 were formed. The products from S2(e) Fig were formed via a combination of Michael-type addition and Schiff base reaction. The UV-vis data exhibited two maxima in absorbance around *λ* = 265 nm and 384 nm.

**Fig 10:**
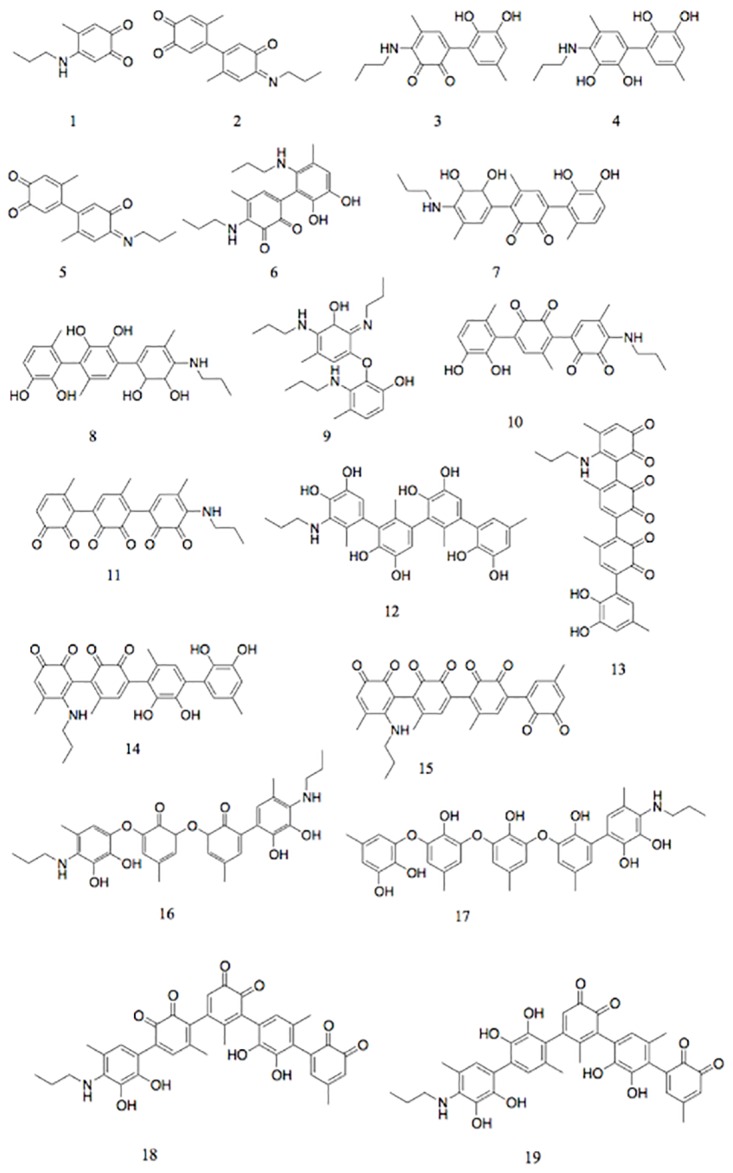
An overview of the proposed structures of the products. The number under each structure corresponds to the number in S1 Table.

Similarly, for all the major peaks in the chromatogram, we have identified the mass spectra, UV-vis spectra and the existence of possible isomers. From this, we construct a list of hypothetical structures for these products. All the details are listed in S3–S5 Figs. Finally, we scanned over the whole HPLC chromatogram, and identified around 60 products in addition to the major peaks (S1 Table and Fig 10), which are associated with small peaks in the HPLC chromatogram.

### Proposed reaction mechanisms of 4MC and PA

Using the assignments from LC-MS, we found that the reaction between 4MC and PA under the current reaction conditions is very fast and many products are formed. Within 5 min, more than 60 products covering the mass range from molecular weight 179 to 704 were identified. These products are categorized into 19 groups based on their mass (S1 Table and Fig 10). It is clear that for most of the products, more than one isomer was formed, which indicates the complexity of the mechanism. The proposed structures in Fig 10 reveal that the majority of the products were formed via aroxyl phenol coupling and Michael-type addition. Only a very small portion was formed by Schiff base reaction. The reason for the formation of limited amount of Schiff base may be twofold. Firstly, to form a Schiff base by a carbonyl group and a primary amine, the first step is the protonation of the carbonyl group to make the carbon more electrophilic. The protonation requires a high enough concentration of protons, which is not fulfilled for our reaction conditions at pH ~ 11. Secondly, the mobile phase for HPLC is a combination of acetonitrile and water containing 0.1% formic acid, which is very acidic. This acidic environment may hydrolyze most of the Schiff base if any has been formed[23].

Using HPLC, we investigated the time dependence of the reaction between 4MC and PA (Fig 7). As time progressed, most of the multiple HPLC peaks that had appeared after 5 min decreased with time, except for two peaks that eluted at retention times of 3.1 and 7.0 min. The intensity of these two peaks increased over 24 h, indicating net formation of these two products. From the identification of MS, these two products showed signals at m/z of 578 (retention time 3.1 min) and 230 (7.0 min), respectively. The mass of m/z = 578 corresponds to compound 15 shown in Fig 7, while the structure corresponding to m/z = 230 remains unidentified. Finally, from the observation that the increasing trend of the two peaks at 3.1 and 7.0 min coincides with a decreasing trend of all other major peaks, we infer a hypothetical pathway of the reaction between 4MC and PA. Upon mixing 4MC with PA in the presence of NaIO_4_, 4MC was first quickly oxidized to *o*-quinone. The highly reactive *o*-quinone either formed crosslinks with catechol, or reacted with nucleophilic amines in PA to form low molecular weight adducts. For instance, PMB was formed after 5 min of reaction, and due to its instability, it disappeared already after one hour of reaction. The low molecular weight intermediates react further with *o*- quinone or PA or other intermediates to form higher molecular weight products. In the course of time, the coupling of these products resulted in low concentrations of less soluble polymeric structures.

**Fig 11:**
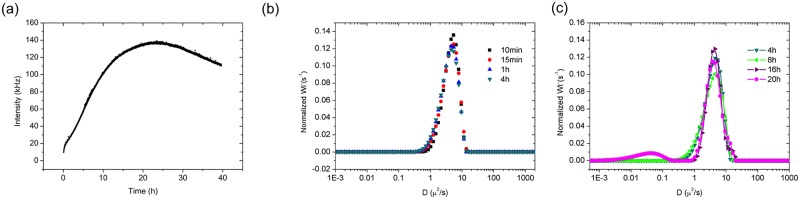
DLS measurement of the reaction system as a function of time: a) Intensity as a function of time, b) the diffusion coefficient as a function of time within 4 h, c) the diffusion coefficient as a function of time within 24 h.

To verify this hypothesis, we performed dynamic light scattering to elucidate more details of this mechanism. Fig 11(a) shows the scattered intensity of the reaction mixture as a function of time. Generally, larger aggregates scatter more light than small ones. From Fig 11(a), it is clear that the intensity initially increased significantly, reaching a maximum around 24 h. This increase in intensity suggests the formation of large aggregates when the reaction proceeds. These large aggregates, in turn, might be due to the formation of high molecular weight poorly soluble adducts from the reaction of 4MC and PA. The formation of large aggregates is also verified by the diffusion coefficient of the reaction mixture over time, as shown in Fig 11(b) and 11(c). As the reaction progressed, the diffusion coefficient gradually shifted to lower values, indicating the formation of larger aggregates. After 16 h, two populations of aggregates with very different sizes, as indicated by the different diffusion coefficients, were formed. The new population of slowly diffusing aggregates arises most likely as a result of physical aggregation of the least soluble reaction products. This verifies our hypothesis that the low molecular weight intermediates gradually transformed into high molecular weight products. After 24 h, there is a decrease in intensity as shown in Fig 11(a). This is likely due to sedimentation of the large aggregates.

## Conclusions

In this study, we have investigated the crosslinking chemistry of catechol and primary amine using model compounds 4MC and PA by spectroscopic and chromatographic methods. We carried out the reaction of 4MC and PA at 20°C in the presence of NaIO_4_ at high pH using 10 mM Na_2_CO_3_ aqueous solution. By separating and identifying the products using HPLC-MS, we found that the crosslinking chemistry of catechol and amine is both fast and complicated. Within five minutes, more than 60 products were formed. These products encompass 19 masses ranging from 179 to 704. The majority of these products are formed via aryloxyl phenol coupling and Michael-type addition; a small fraction is formed via Schiff base reactions.

Our findings highlight the major reaction pathways and time scales for the reactions between catechols and amines. This result can be used as a guideline to design crosslinking strategies and material properties. More specifically, by controlling the reaction conditions, e.g. pH, it is possible to control the type of products formed. For materials that need chemical stability under different pH values, the formation of catechol-catechol coupling or Michael type adduct is favored. In contrast, the formation of Schiff base should be minimized due to its sensitivity to hydrolysis, which can affect the properties of the materials. Moreover, these findings aid to a better understanding of the catechol crosslinking chemistry in natural and synthetic systems.

## Supporting Information

S1 Fig ^1^H NMR spectra monitoring the reaction of 4MC and NaIO_4_ in 10 mM Na_2_CO_3_/D_2_O with time. The molar ratio between 4MC and NaIO_4_ is 0.5. Dioxane was used as a reference. The enlarged peaks at 1.5 ppm were marked in red box.
(EPS)

S2 Fig a) LC-MS chromatograms (monitored at 254 nm) of the reaction mixture of 4MC, PA, and NaIO_4_ over time. The ratio of 4MC, PA and NaIO_4_ is 1:3:0.5. b) Extracted ion chromatogram (positive ion mode) corresponding to product with m/z = 439.5–440.5 c) ESI-MS spectra [m/z 50–2000] summed over the 11.00–11.10 min retention time window; d) Extracted UV spectra corresponding to the 11.00–11.10 min retention time e) Proposed representative chemical structure of product.
(EPS)

S3 Fig a) LC-MS chromatograms (monitored at 254 nm) of the reaction mixture of 4MC, PA, and NaIO_4_ over time. The ratio of 4MC, PA and NaIO_4_ is 1:3:0.5. b) Extracted ion chromatogram (positive ion mode) corresponding to m/z = 337.5–338.5 c) ESI-MS spectra [m/z 50–2000] summed over the 5.93–6.15 min retention time window; d) Extracted UV spectra corresponding to the 5.93–6.15 min retention time e) Proposed representative chemical structure of product.
(EPS)

S4 Fig a) LC-MS chromatograms (monitored at 254 nm) of the reaction mixture of 4MC, PA, and NaIO_4_ over time. The ratio of 4MC, PA and NaIO_4_ is 1:3:0.5. b) Extracted ion chromatogram (positive ion mode) corresponding to m/z = 425.5–426.5 c) ESI-MS spectra [m/z 50–2000] summed over the 8.6–8.91 min retention time window; d) Extracted UV spectra corresponding to the 8.6–8.91 min retention time e) Proposed representative chemical structure of product.
(EPS)

S5 Fig a) LC-MS chromatograms (monitored at 254 nm) of the reaction mixture of 4MC, PA, and NaIO_4_ over time. The ratio of 4MC, PA and NaIO_4_ is 1:3:0.5. b) Extracted ion chromatogram (positive ion mode) corresponding to m/z = 423.5–424.5 c) ESI-MS spectra [m/z 50–2000] summed over the 8.70–9.20 min d) Extracted UV spectra corresponding to the 8.70–9.20 min retention time e) Proposed representative chemical structure of product.
(EPS)

S6 Fig Proposed mechanism of reaction between 4-methyl catechol and propylamine by Michael-type addition and phenol coupling. (EPS)

S1 Table Identification of peaks in LC-MS, based on MS and UV-VIS data. (DOCX)
