# Supplementary material for: Reaction Pathways in Catechol/Primary Amine Mixtures: A Window on Crosslinking Chemistry
Source: PLoS One. 2016 Dec 8;11(12):e0166490. doi: 10.1371/journal.pone.0166490 (PMC5145154; doi:10.1371/journal.pone.0166490)
Supplement: S1 Table — (DOCX) [file pone.0166490.s007.docx]

**S1 Table.** Identification of peaks in LC-MS, based on MS and UV-VIS data

| *Entry* | *Retention time (min)* | *M/Z* | *Ion type* | *UV(λ_max_)* | *Product number* | *n_1_* | *n_2_* | *n_3_* | *n_4_* | *n_5_* |
| --- | --- | --- | --- | --- | --- | --- | --- | --- | --- | --- |
| *1* | *17.86* | *180* | *[M+H]^+^* | *236,272,355* | *1* | *0* | *1* | *1* | *1* | *0* |
| *2* | *13.19* | *261* | *[M+Na]^+^* | *235,281* | *2* | *0* | *1* | *2* | *1* | *1* |
| *3* | *15.06* | *261* | *[M+Na]^+^* | *235,265(329)* | *2* | *0* | *1* | *2* | *1* | *1* |
| *4* | *14.62* | *302* | *[M+H]^+^* | *235,285* | *3* | *1* | *1* | *1* | *2* | *0* |
| *5* | *18.61* | *302* | *[M+H]^+^* | *236,330,441* | *3* | *1* | *1* | *1* | *2* | *0* |
| *6* | *9.57* | *304* | *[M+H]^+^* | *234* | *4* | *2* | *1* | *0* | *1* | *0* |
| *7* | *10.41* | *304* | *[M+H]^+^* | *234,276* | *4* | *2* | *1* | *0* | *1* | *0* |
| *8* | *10.56* | *304* | *[M+H]^+^* | *234,264* | *4* | *2* | *1* | *0* | *1* | *0* |
| *9* | *14.73* | *304* | *[M+H]^+^* | *235,281* | *4* | *2* | *1* | *0* | *1* | *0* |
| *10* | *17.82* | *304* | *[M+H]^+^* | *236,267,353* | *4* | *2* | *1* | *0* | *1* | *0* |
| *11* | *6.31* | *322* | *[M+K]^+^* | *234* | *5* | *0* | *1* | *2* | *1* | *1* |
| *12* | *7.24* | *322* | *[M+K]^+^* | *234,338* | *5* | *0* | *1* | *2* | *1* | *1* |
| *13* | *9.52* | *322* | *[M+K]^+^* | *234* | *5* | *0* | *1* | *2* | *1* | *1* |
| *14* | *9.69* | *322* | *[M+K]^+^* | *234,278,309* | *5* | *0* | *1* | *2* | *1* | *1* |
| *15* | *10.27* | *359* | *[M+H]^+^* | *235,294,504* | *6* | *1* | *2* | *1* | *3* | *0* |
| *16* | *22.68* | *359* | *[M+H]^+^* | *236* | *6* | *1* | *2* | *1* | *3* | *0* |
| *17* | *8.97* | *424* | *[M+H]^+^* | *235,324* | *7* | *2* | *1* | *1* | *3* | *0* |
| *18* | *15.36* | *424* | *[M+H]^+^* | *235,264,355,384* | *7* | *2* | *1* | *1* | *3* | *0* |
| *19* | *8.66* | *426* | *[M+H]^+^* | *235* | *8* | *3* | *1* | *0* | *3* | *0* |
| *20* | *9.06* | *426* | *[M+H]^+^* | *234* | *8* | *3* | *1* | *0* | *3* | *0* |
| *21* | *12.38* | *426* | *[M+H]^+^* | *235* | *8* | *3* | *1* | *0* | *3* | *0* |
| *22* | *10.04* | *440* | *[M+K]^+^* | *234* | *9* | *2* | *3* | *0* | *3* | *1* |
| *23* | *17.98* | *440* | *[M+K]^+^* | *236,283,347* | *9* | *2* | *3* | *0* | *3* | *1* |
| *24* | *19.33* | *440* | *[M+K]^+^* | *236,281,382* | *9* | *2* | *3* | *0* | *3* | *1* |
| *25* | *20.22* | *440* | *[M+K]^+^* | *236* | *9* | *2* | *3* | *0* | *3* | *1* |
| *26* | *9.23* | *444* | *[M+Na]^+^* | *234* | *10* | *1* | *1* | *2* | *3* | *0* |
| *27* | *9.89* | *444* | *[M+Na]^+^* | *234* | *10* | *1* | *1* | *2* | *3* | *0* |
| *28* | *13.76* | *444* | *[M+Na]^+^* | *235* | *10* | *1* | *1* | *2* | *3* | *0* |
| *29* | *14.39* | *444* | *[M+Na]^+^* | *235* | *10* | *1* | *1* | *2* | *3* | *0* |
| *30* | *6.86* | *458* | *[M+K]^+^* | *234,276* | *11* | *0* | *1* | *3* | *3* | *0* |
| *31* | *10.04* | *458* | *[M+K]^+^* | *234* | *11* | *0* | *1* | *3* | *3* | *0* |
| *32* | *16.43* | *458* | *[M+K]^+^* | *235(354)* | *11* | *0* | *1* | *3* | *3* | *0* |
| *33* | *17.55* | *458* | *[M+K]^+^* | *236(346)* | *11* | *0* | *1* | *3* | *3* | *0* |
| *34* | *13.71* | *548* | *[M+H]^+^* | *235* | *12* | *4* | *1* | *0* | *4* | *0* |
| *35* | *18.61* | *548* | *[M+H]^+^* | *236,333,441* | *12* | *4* | *1* | *0* | *4* | *0* |
| *36* | *19.89* | *548* | *[M+H]^+^* | *236,346* | *12* | *4* | *1* | *0* | *4* | *0* |
| *37* | *20.81* | *548* | *[M+H]^+^* | *236,350* | *12* | *4* | *1* | *0* | *4* | *0* |
| *38* | *12.3* | *564* | *[M+Na]^+^* | *235* | *13* | *1* | *1* | *3* | *4* | *0* |
| *39* | *17.84* | *564* | *[M+Na]^+^* | *236,268,355* | *13* | *1* | *1* | *3* | *4* | *0* |
| *40* | *19.1* | *564* | *[M+Na]^+^* | *236,346* | *13* | *1* | *1* | *3* | *4* | *0* |
| *41* | *19.44* | *564* | *[M+Na]^+^* | *236,281,399* | *13* | *1* | *1* | *3* | *4* | *0* |
| *42* | *11.09* | *566* | *[M+Na]^+^* | *233,265,384* | *14* | *2* | *1* | *2* | *4* | *0* |
| *43* | *17.82* | *566* | *[M+Na]^+^* | *236,267,353* | *14* | *2* | *1* | *2* | *4* | *0* |
| *44* | *17.65* | *578* | *[M+K]^+^* | *236* | *15* | *0* | *1* | *4* | *4* | *0* |
| *45* | *17.97* | *578* | *[M+K]^+^* | *236,347* | *15* | *0* | *1* | *4* | *4* | *0* |
| *46* | *19.48* | *578* | *[M+K]^+^* | *236,283,396* | *15* | *0* | *1* | *4* | *4* | *0* |
| *47* | *19.56* | *578* | *[M+K]^+^* | *236,349* | *15* | *0* | *1* | *4* | *4* | *0* |
| *48* | *14.28* | *605* | *[M+H]^+^* | *235* | *16* | *2* | *2* | *2* | *5* | *0* |
| *49* | *14.53* | *605* | *[M+H]^+^* | *235* | *16* | *2* | *2* | *2* | *5* | *0* |
| *50* | *16.17* | *605* | *[M+H]^+^* | *235(352)* | *16* | *2* | *2* | *2* | *5* | *0* |
| *51* | *17.93* | *670* | *[M+H]^+^* | *236,352* | *17* | *5* | *1* | *0* | *5* | *0* |
| *52* | *19.01* | *670* | *[M+H]^+^* | *236,346* | *17* | *5* | *1* | *0* | *5* | *0* |
| *53* | *20.35* | *670* | *[M+H]^+^* | *236(346)* | *17* | *5* | *1* | *0* | *5* | *0* |
| *54* | *16.5* | *702* | *[M+K]^+^* | *235(352)* | *18* | *2* | *3* | *1* | *5* | *0* |
| *55* | *16.96* | *702* | *[M+K]^+^* | *236(346)* | *18* | *2* | *3* | *1* | *5* | *0* |
| *56* | *18.36* | *702* | *[M+K]^+^* | *236(354)* | *18* | *2* | *3* | *1* | *5* | *0* |
| *57* | *10.95* | *704* | *[M+K]^+^* | *234,264* | *19* | *3* | *1* | *2* | *5* | *0* |
| *58* | *11.48* | *704* | *[M+K]^+^* | *235,276* | *19* | *3* | *1* | *2* | *5* | *0* |
| *59* | *17.07* | *704* | *[M+K]^+^* | *236(346)* | *19* | *3* | *1* | *2* | *5* | *0* |
| *60* | *16.96* | *704* | *[M+K]^+^* | *236(346)* | *19* | *3* | *1* | *2* | *5* | *0* |
